# Estimating population extinction thresholds with categorical classification trees for Louisiana black bears

**DOI:** 10.1371/journal.pone.0191435

**Published:** 2018-01-23

**Authors:** Jared S. Laufenberg, Joseph D. Clark, Richard B. Chandler

**Affiliations:** 1 Department of Forestry, Wildlife and Fisheries, University of Tennessee, Knoxville, Tennessee, United States of America; 2 U.S. Geological Survey, Southern Appalachian Research Branch, Northern Rocky Mountain Science Center, University of Tennessee, Knoxville, Tennessee, United States of America; 3 Warnell School of Forestry and Natural Resources, University of Georgia, Athens, Georgia, United States of America; Université de Sherbrooke, CANADA

## Abstract

Monitoring vulnerable species is critical for their conservation. Thresholds or tipping points are commonly used to indicate when populations become vulnerable to extinction and to trigger changes in conservation actions. However, quantitative methods to determine such thresholds have not been well explored. The Louisiana black bear (*Ursus americanus luteolus*) was removed from the list of threatened and endangered species under the U.S. Endangered Species Act in 2016 and our objectives were to determine the most appropriate parameters and thresholds for monitoring and management action. Capture mark recapture (CMR) data from 2006 to 2012 were used to estimate population parameters and variances. We used stochastic population simulations and conditional classification trees to identify demographic rates for monitoring that would be most indicative of heighted extinction risk. We then identified thresholds that would be reliable predictors of population viability. Conditional classification trees indicated that annual apparent survival rates for adult females averaged over 5 years (${\bar{\varphi }}_{5}$) was the best predictor of population persistence. Specifically, population persistence was estimated to be ≥95% over 100 years when ${\bar{\varphi }}_{5}\geq 0.90$, suggesting that this statistic can be used as threshold to trigger management intervention. Our evaluation produced monitoring protocols that reliably predicted population persistence and was cost-effective. We conclude that population projections and conditional classification trees can be valuable tools for identifying extinction thresholds used in monitoring programs.

## Introduction

Ecological monitoring is an important component of natural resource management and conservation of biodiversity [1]. In recent years, monitoring-related topics such as their purpose and need [2–6], design and implementation [7–9], and shortcomings and pitfalls [10,11] have received considerable attention. A successful monitoring program can be a complex and extensive undertaking often requiring substantial investment of time and resources throughout program inception, design, implementation, and assessment.

Monitoring threatened or endangered species during the recovery phase often focuses on factors such as population response to change in habitat quantity and quality, abatement of risks to the target species, or overall assessment of population status relative to recovery criteria. Because recovery criteria should be based on clearly justified demographic targets meant to alleviate threats to long-term persistence [12], recovery monitoring often requires substantial resources to collect the requisite data to meet those standards. Once recovery occurs, species monitoring should continue for a period of time, preferably using methods consistent with and comparable to monitoring prior to recovery [13]. However, monitoring may be less intense if the effort put forth can reliably detect deterioration of a species’ status. One such approach to reducing post-recovery monitoring effort is to monitor indicators of population status rather than the original criteria used during recovery which, when one or more fall below or rise above a specified threshold, would indicate the need for intervention [13,14]. Such demographic thresholds or triggers can be effective components of population monitoring plans when relationships between demographic rate values and some measure of population vigor can be reliably estimated [14]. For example, survival might serve as a useful index of population persistence probability for which some minimum tipping point could be established. Thus, objectives and constraints of monitoring recovered species are different from those for imperiled species and monitoring programs should adapt to such changes.

In 1992, the U.S. Fish and Wildlife Service (USFWS) granted the Louisiana black bear (*Ursus americanus luteolus*) threatened status under the U.S. Endangered Species Act (ESA), listing loss and fragmentation of habitat as the primary threats [15]. The 1995 Recovery Plan outlined recovery goals designed to meet the objective of reducing threats to the Louisiana black bear and the habitat supporting it [16]. Laufenberg et al. [17] performed a population viability analysis (PVA) for the Louisiana black bear based in part on capture-mark-recapture (CMR) data collected in the Tensas River Basin (TRB) from 2006 to 2012 and the Upper Atchafalaya River Basin (UARB) from 2007 to 2012. The CMR data consisted of genotyped bear hair samples collected at barbed wire sampling sites. These data were used to develop stochastic population models using hierarchical Bayesian modeling methods to estimate probability of persistence. Laufenberg et al. [17] concluded that the probability of persistence of the TRB and UARB subpopulations over 100 years were >0.928 and >0.906, respectively, which helped support the decision to remove *U*. *a*. *luteolus* from the list of threatened species in March 2016 [18].

A post-delisting monitoring plan was developed and implemented by the Louisiana Department of Wildlife and Fisheries (LDWF) and the USFWS [19]. As is the case with many post-recovery monitoring plans, it did not include the effects of temporal stochasticity on population trend or explicitly link indicators of population status and indicator thresholds to persistence that would trigger a change in Louisiana black bear conservation status. Thus, our objectives were to explore methods to identify indicators of population persistence and determine monitoring thresholds that were data based, robust, included parameter uncertainty, and incorporated environmental variation, using the Louisiana black bear as a case study.

## Methods

### General approach

Our general approach was comprised of 2 stages. First, we conducted a sensitivity analysis using stochastic population simulations combined with conditional classification trees to identify demographic rates most important to long-term extinction risk for bears of the TRB and UARB subpopulations. The second stage was to identify short-term demographic parameters that could be used as indicators of long-term population persistence (i.e., thresholds) at the TRB and the UARB that could be estimated by modifying existing data collection protocols.

### Sensitivity analysis

To perform a sensitivity analysis, it was first necessary to develop a population projection model for calculating extinction rates. The CMR estimates used by Laufenberg et al. [17] were based on the use of molecular markers to obtain unique, multilocus genotypes of individual animals [20]. To ensure that all bears would have opportunities to be sampled, hair collection sites were spaced so that ≥4 sites would be available per adult female home range [21]. The complete CMR data set used by Laufenberg et al. [17] consisted of DNA-based binary detection records (i.e., 1 if detected and 0 if not) of individual bears obtained from hair collection surveys conducted across arrays of hair collection sites in the TRB and UARB populations. Surveys were conducted in a robust-design format consisting of primary sampling occasions (i.e., years) between which the population was considered open to gains and losses and secondary occasions (i.e., weeks) within primary occasions during which the population was considered geographically and demographically closed [22].

Laufenberg et al. [17] used a hierarchical CMR modeling framework based on a state-space parameterization of the Jolly-Seber model [23,24] to estimate abundance (*N*), annual apparent survival (*φ*), annual per-capita recruitment (*f*), annual realized population rate-of-change (*λ*), and weekly detection probabilities (*p*). To separate sampling variance from process variance for *φ* and *f*, annual values for each of those vital rates were treated as random variables coming from a common hyperdistribution using an appropriate link function. The hyperdistribution for *φ* was logit (*φ*) = *μ*_*φ*_ + *ε*_*t*_ where *ε*_*t*_ ~ Normal (0, *σ*_*φ*_^2^) and *μ*_*φ*_ was the overall mean annual apparent survival on the logit scale, *ε*_*t*_ was the annual deviation from the mean in year *t*, and *σ*_φ_^2^ was the temporal process variance. Temporal process variation of *f* (*σ*_*f*_^2^) was similarly modeled. To model density dependence in per-capita recruitment [25], we defined a log-linear model for the relationship between *f* and *N* was defined as log (*f*) = *β*_*0f*_ *+ β*_*1f*_*N*_*t*_ + *ε*_*t*_, where *ε*_*t*_ ~ Normal (0, *σ*_*f*_^2^) and *β*_*0f*_ and *β*_*1f*_ were the intercept and slope parameters, respectively. Thus, demographic rates used in the projection model included *μ*_*φ*_, *σ*_*φ*_ (standard deviation of *σ*_*φ*_^2^), *β*_*0f*_, *β*_*1f*_, and *σ*_*f*_ (standard deviation of *σ*_*f*_^2^). Apparent survival (*φ*) included mortalities and emigrants. Estimates of *f* included births and immigrants and, when summed with *φ*, produced the finite population growth rate (*λ*), which represented the annual realized rate of change in abundance as a function of births, deaths, immigration, and emigration.

We adopted a Bayesian approach to population viability analysis based on stochastic population projection methods to assess probability of persistence. A population projection simply is 1 of many possible population trajectories, some of which are more likely to occur than others based on a stochastic model with a number of simplifying assumptions that govern population dynamics. Probability of persistence can be inferred from those trajectory outcomes most likely to occur (i.e., extinction vs. persistence) while accounting for uncertainty caused by stochastic population processes. Our goal was to develop a set of models based on a range of biologically reasonable model parameters and assumptions to project subpopulation trajectories and characterize persistence probabilities.

We used Monte Carlo methods to include 2 types of random variation into each model: temporal (environmental) stochasticity in annual vital rates and demographic stochasticity in individual life-history events. One goal of the sensitivity analysis was to simulate population trajectories across a much wider range of demographic conditions (i.e., different combinations of *φ* and *f*) than did Laufenberg et al. [17]. We wanted to relax the assumption made in the original PVA that the population dynamics (i.e., means and variances) observed during that study would remain stationary for the determination of long-term viability to be valid. Thus, we randomly sampled individual parameter values for each trajectory from uniform distributions, defined by the minimum and maximum values of the posterior distribution for each parameter reported in Laufenberg et al. [17] (Table 1), rather than directly from the respective parameter posterior distributions. We also independently sampled each parameter value instead of sampling from the joint posterior distribution as was done in Laufenberg et al. [17]. This produced a data set that included biologically plausible combinations of parameter values not observed during the PVA study, which allowed us to broaden our inferences for individual population parameters most associated with extinction risk and identify demographic thresholds indicative of long-term persistence.

**Table 1:** Minimums and maximums of posterior distributions for demographic rates estimated by Laufenberg et al. (2016) from DNA-based capture-mark-recapture data collected from Louisiana black bears in the Tensas River Basin (TRB; 2006–2012) and in the Upper Atchafalaya River Basin (UARB; 2007–2012), Louisiana, USA. Recruitment parameters were estimated on the natural log scale and survival parameters on the logit scale.

	TRB		UARB	
	Minimum	Maximum	Minimum	Maximum
Recruitment intercept (*β*_*0f*_)^1^	-3.63	42.59	-3.96	43.27
Recruitment slope (*β*_*1f*_)	-0.33	-3.40 × 10^−5^	-1.34	-1.70 × 10^−6^
Recruitment standard deviation (σ_*f*_)	6.33 × 10^−5^	2.00	1.06 × 10^−5^	2.00
Survival mean (*μ*_*φ*_)^2^	0.85	3.68	0.35	4.10
Survival standard deviation (σ_*φ*_)	1.33 × 10^−4^	1.00	8.59 × 10^−5^	1.00

^1^Recruitment parameters estimated on the natural log scale.

^2^Survival parameters estimated on the logit scale.

For each projection model, we simulated trajectories over a 100-year period. We generated 2,000 random combinations of projection model parameters from the above uniform distributions for the TRB and 2,000 for the UARB. We then used each of those combinations to assess stochasticity by simulating 500 population trajectories for 100 years resulting in 1,000,000 trajectories for each subpopulation. For each trajectory, we recorded the model parameter values used in the projection (*μ*_*φ*_, *σ*_*φ*_, *β*_*0f*_, *β*_*1f*_, and *σ*_*f*_) and annual values of *λ*, *φ*, *f*, and *N*.

To identify the most important parameter values for predicting extinction risk, we chose recursive partitioning, a widely used method for nonparametric regression and classification. Recursive partitioning is a multivariable analytical method that creates a decision tree that attempts to classify members of the population by splitting them into subpopulations based on dichotomous independent variables. A type of recursive partitioning is known as random forests, which has been successfully applied in many scientific fields [26]. Random forests employs conditional inference trees, which is a non-parametric class of regression trees based on machine learning [27]. To perform the analysis we first created a binary response variable (EXTANT) by assigning a value of 1 to each simulated trajectory with the number of bears in the population after 100 years (*N*_*100*_) ≥ 1 and assigned a value of 0 otherwise. We then randomly selected 500,000 of the 1,000,000 simulated trajectories as a training data set for which we constructed random forests of conditional classification trees for the TRB and the UARB using the cforest function in the R package party [28–30]. Explanatory variables included all 5 data-generating parameter values (*μ*_*φ*_, σ_*φ*_, *β*_*0*_, *β*_*1*_, and σ_*f*_). We restricted the size of individual trees by limiting tree depth to 4 levels to ensure interpretability of individual trees and to reduce suboptimal performance associated with overfitting when sample size is large and the number of explanatory variables is small [31]. We set the number of explanatory variables randomly selected for determining individual splits at 5, the maximum number of available explanatory variables in our data set, and set the number of trees in the forest to 100 which we determined was sufficient for reliable prediction and assessment of variable importance given the relatively small number of explanatory variables while minimizing computational burden. All other function arguments were left at default values. Variable importance scores were calculated using the varimp function in the party package. We assessed model fit and predictive performance of the random forests by calculating the overall classification error rate and the error rate for incorrectly classifying a trajectory that went extinct as extant (i.e., Type II error rate). We performed those calculations for a holdout sample independent of the training data set that was comprised of 10,000 trajectories randomly selected from those trajectories not selected for the training data set. We considered Type II error (i.e., wrongly predicting persistence) to be the most appropriate for endangered species decision making.

In addition to the overall analysis of the relationship between individual population parameters and extinction risk, we also wanted to evaluate demographic parameters estimated over a time span more appropriate for a typical post-recovery monitoring period (e.g., 5 years). To evaluate which demographic parameters estimated over a 5-year time span best predicted extinction risk, we derived new variables based on values for *N*, *φ*, and *λ* extracted from the training data set. We calculated the mean value for each variable observed during the first 5 (${\bar{N}}_{5}$, ${\bar{\varphi }}_{5}$, and ${\bar{\lambda }}_{5}$) years of each population projection and used those explanatory variables in separate random forests analyses each for the TRB and UARB. We used the same function settings and assessments of model fit and predicted performance as we did for the overall analysis except that we set the number of explanatory variables selected for determining splits to 3, the maximum number of available explanatory variables in these data sets.

### Demographic thresholds

Traditional statistical analysis methods (e.g., generalized linear models or analyses of variance) can be inadequate for revealing complex nonlinear relationships in high-dimensional ecological data, whereas machine-learning methods such as classification trees are well suited to such situations [32,33]. We used single conditional-inference classification tree analysis to explore relationships and identify robust predictive thresholds between demographic rates and the likelihood of population extinction.

We were primarily interested in identifying demographic rate thresholds that would be reliable indicators of long-term population persistence (i.e., persistence probability ≥95%) and could be measured over relatively short monitoring durations (e.g., 5 years). Therefore, we constructed conditional classification trees for the TRB and UARB populations based on demographic rates consistent with our sensitivity analysis over a 5-year monitoring duration. The classification tree was focused on duration-specific average values derived from annual demographic rates generated by our population simulations. Those averages were defined based on rate-specific numeric scales where *N* and *λ* could range from 0 to ∞ and *φ* could range from 0 to 1.

We used the same training data sets and explanatory variables as our variable importance analysis and the ctree function in the R package party [34] to grow separate classification trees for each set of predictor variables for the TRB and UARB. We converted the EXTANT variable from the variable importance data set from a binary variable to a factor variable with 2 levels, EXTINCT when *N*_*100*_ <1 and EXTANT otherwise, to accommodate the data structure required by the ctree function. Again, we restricted the size of the trees to 4 levels, set the number of explanatory variables selected for determining splits to 3, and set the number of trees in the forest to 100; all other function arguments were left at default values. We assessed model fit and predictive performance based on a hold-out sample of 10,000 trajectories independent of the training data set as we did for the random forests analysis. We defined reliable demographic rate thresholds as scenarios (i.e., classification tree branches) from our classification trees that resulted in persistence probabilities ≥95% for 100 years.

## Results

**Fig 1:**
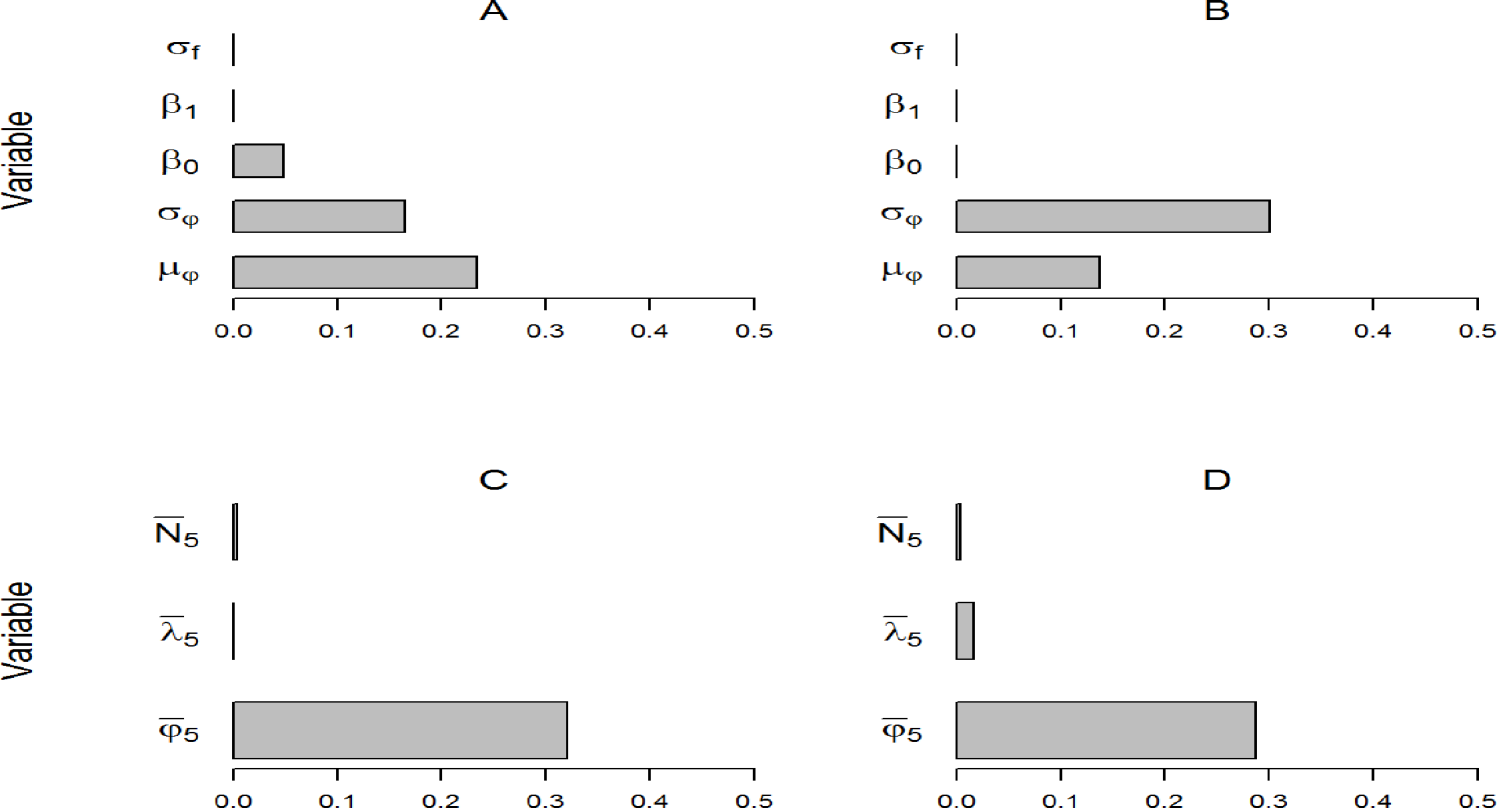
Variable importance scores (on the x axes) estimated from random forests of conditional classification trees based on stochastic population simulations for females in the Tensas River Basin (panels A and C) and Upper Atchafalaya River Basin (panels B and D) subpopulations. Explanatory variables corresponded to long-term demographic rates used to generate population trajectories (panels A and B) or averages derived from the first 5 years (panels C and D). Variables were temporal variation in per-capita recruitment (σ_*f*_), the intercept and slope coefficients describing log-linear density-dependence in per-capita recruitment (*β*_*0f*_ and *β*_*1f*_), temporal variation in apparent survival (σ_*φ*_), mean apparent survival ($\mu_{\varphi }$), average abundance over a 5-year period (${\bar{N}}_{5}$), average population growth rates over a 5-year period (${\bar{\lambda }}_{5}$), and average apparent survival probabilities over 5 years (${\bar{\varphi }}_{5}$).

Mean annual female *φ* on the logit scale (*μ*_*φ*_) was identified as the long-term rate with the greatest relative importance for predicting extinction of the TRB subpopulation (Fig 1A). Annual variation of *φ* (σ_*φ*_) was also a strong predictor for the TRB. Conversely, σ_*φ*_ had the greatest relative importance to extinction risk and *μ*_*φ*_ was only moderately important in the UARB (Fig 1B). The overall classification error rate for the random forests model predicting extinction in the TRB was 12.4% and the Type II error rate (i.e., predicted outcome is EXTANT when true outcome is EXTINCT) was 7.5% indicating reasonable predictive power. The random forests model for the UARB performed better than for the TRB with an overall error rate of 5.9% and Type II error rate of 3.2%.

Of the short-term rates evaluated for the TRB and UARB, average apparent female survival (${\bar{\varphi }}_{5}$) was most important for predicting extinction for both subpopulations (Fig 1C and 1D). Overall and Type II error rates for the TRB random forests based a 5-year duration were 8.5% and 3.2%. For the UARB, overall and Type II error rates were 8.6% and 3.7%, respectively.

**Fig 2:**
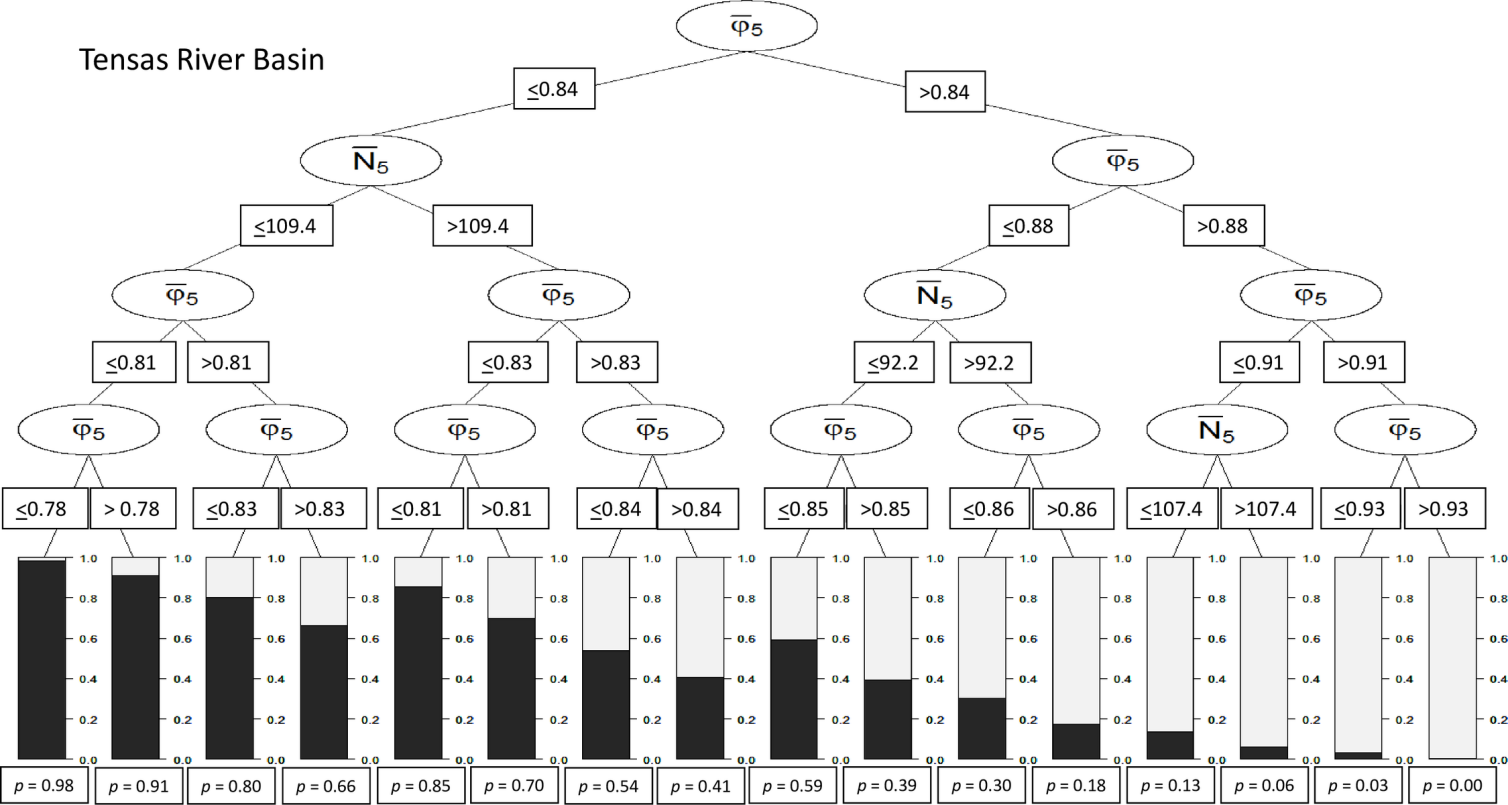
Conditional classification tree for short-term female demographic thresholds for the for the Tensas River Basin subpopulation based on a 5-year monitoring duration. Proportions of bars in dark gray and values below bars (e.g., *p* = 0.98) represent extinction probability. Variables were average abundance over a 5-year period (${\bar{N}}_{5}$) and average apparent survival probabilities over 5 years (${\bar{\varphi }}_{5}$).

**Fig 3:**
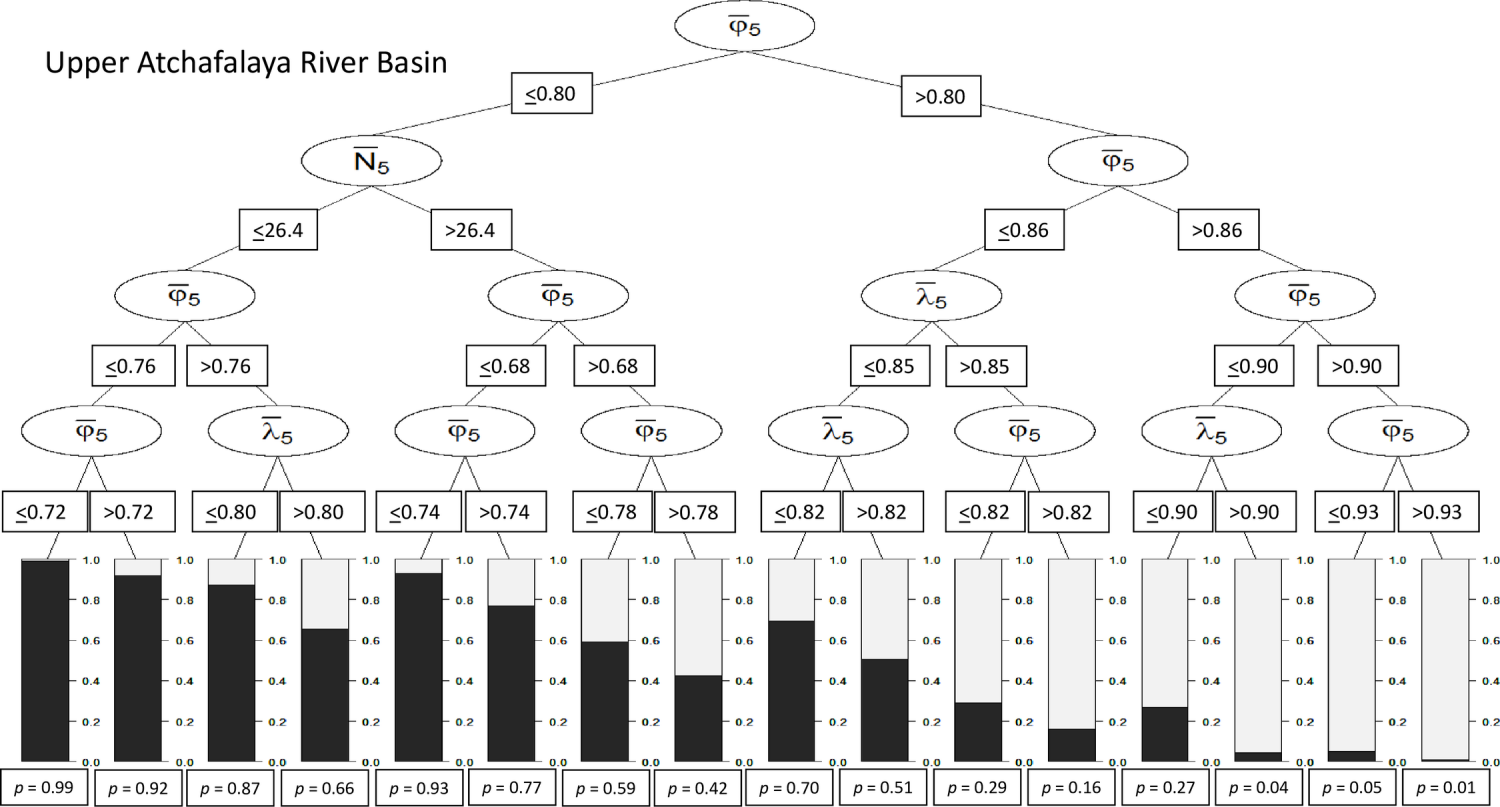
Conditional classification tree for short-term female demographic thresholds for the Upper Atchafalaya River Basin subpopulation based on a 5-year monitoring duration. Proportions of bars in dark gray and values below bars (e.g., *p* = 0.99) represent extinction probability. Variables were average abundance over a 5-year period (${\bar{N}}_{5}$), average population growth rates over a 5-year period (${\bar{\lambda }}_{5}$), and average apparent survival probabilities over 5 years (${\bar{\varphi }}_{5}$).

For the TRB subpopulation, 8 combinations of demographic threshold values (hereafter scenarios) were classified as extant (Fig 2). Scenarios for which ${\bar{\varphi }}_{5}$ was >0.91 resulted in likelihoods of ≥95% that the TRB subpopulation would remain extant for 100 years (Fig 2). At UARB, 9 demographic scenarios resulted in probabilities of persistence >0.5 (Fig 3). Of the 3 scenarios with high likelihoods of persistence (i.e., ≥95%) for the 5-year duration, 2 were solely based on thresholds for ${\bar{\varphi }}_{5}$ and the remaining scenario included thresholds for ${\bar{\varphi }}_{5}$ and ${\bar{\lambda }}_{5}$. The minimum threshold for scenarios involving only apparent survival was 0.90 (${\bar{\varphi }}_{5}$) for the 5-year durations, which was nearly identical to the value for the TRB subpopulation.

## Discussion

Our simulations suggest that a threshold of ${\bar{\varphi }}_{5}$ = 0.90 can reliably predict a 95% probability of persistence for both bear subpopulations. Other triggers are possible depending on different probabilities of persistence or levels of uncertainty with which managers are comfortable. The threshold ${\bar{\varphi }}_{5}$ can be estimated using open population temporal symmetry CMR models [35] or Cormack-Jolly-Seber models [36], both of which are relatively robust to common capture biases [37]. Post-hoc simulations using CMR data from UARB suggest that 17 barbed-wire hair-sampling sites checked for 4 weeks each year would reliably estimate *φ*. This scenario represents a significant decrease in monitoring intensity at UARB compared with what was prescribed in the monitoring plan (116 traps sampled for 3 weeks/year)[19].

A better understanding of the demographic rates that drive population dynamics is vital to long-term planning, especially for monitoring persistence, and our sensitivity analysis helped to provide that insight. For example, viability of the UARB subpopulation was most sensitive to variation in annual female *φ*, whereas the TRB subpopulation was most affected by the long-term mean of annual female *φ*. Greater importance of temporal variation to persistence of the UARB is reasonable given its relatively small size which makes it more susceptible to environmental stochasticity, even when long-term average female *λ* is positive [17,38,39]. The importance of female *φ* also was evident in our short-term demographic rate classification trees as it was included in every scenario that resulted in population persistence (≥95%). Error rates of short-term demographic rates were smaller than long-term rates for the TRB, possibly related to lower importance values of the explanatory variables in the former data set relative to the latter. Also, UARB had lower error rates than TRB, likely because the smaller initial abundance used in simulations for UARB caused its likelihood of extinction to be more sensitive to small perturbations in important demographic rates which translated into greater predictive power for those rates.

The conditional classification tree methods that we employed were designed to focus on detecting and describing patterns in the data rather than allowing for insights into ecological process [40], which is consistent with our objective of identifying thresholds to be used in monitoring programs. However, data-mining techniques often reveal patterns in the data that can lend themselves to confirmatory analysis based on observational data [41]. Indeed, the importance of adult female *φ* identified by the classification trees has been reported to be a primary driver of black bear population dynamics based on parametric statistical analyses [42,43,44].

We identified design improvements that would result in monitoring efforts that better track population persistence, were cost-effective, and maintained data collection protocols consistent with recovery monitoring. Discovering such improvements is the primary goal for Phase 4 of the “road map” for designing and implementing a monitoring program developed by Reynolds et al. [9]. The transition from the recovery phase to a post-recovery phase necessitates a reevaluation of monitoring methods to ensure collection of quality information yet maximize cost-effectiveness to sustain an extended monitoring period. Although such an evaluation was conducted prior to recovery and subsequent revisions of recovery monitoring methods were incorporated into the monitoring plan, our analysis demonstrates that multiple iterations of learning and revision outlined in the “road map” may be necessary. Our reevaluation process also aligns with principles of adaptive management in that we updated our model of population dynamics used for our sensitivity analysis with information obtained from the PVA.

Lindenmayer et al. [45] describe monitoring programs whereby species were monitored until they went locally, regionally, or globally extinct, because no pre-specified management intervention was triggered. They recommended that conservation monitoring programs explicitly articulate how monitoring information will inform conservation actions, that trigger points be identified with specific strategic interventions should those points be exceeded, and that the ability to achieve early detection of change be rigorously quantified. Effective monitoring programs must also be adaptive to accommodate changes in management or conservation goals, to incorporate better understanding of system function gained from monitoring activities, and implement new developments in monitoring methods [9,46,47]. Such adaptive capacity ensures monitoring programs continue to provide reliable information for making evidence-based management and conservation decisions within an effective and efficient framework. The analytical methods we describe here are statistically rigorous, incorporate parameter uncertainty and environmental variance, and can be used as a tool to evaluate the effect of alternative management actions on population viability consistent with principles of adaptive management. Regardless, we suggest that managers should look at the entirety of the data available for species monitoring rather than rely on a single go, no-go tipping point for any one subpopulation. Although our data set was comprehensive, any data set used to project population growth and estimate extinction risks should be amenable to the use of classification trees to evaluate sensitivity and identify short-term thresholds.
