# Correction: Estimating population extinction thresholds with categorical classification trees for Louisiana black bears

**DOI:** 10.1371/journal.pone.0198368

**Published:** 2018-05-24

**Authors:** The PLOS ONE Staff

There is an error in the Acknowledgements section. The paragraph “This draft manuscript is distributed solely for purposes of scientific peer review. Its content is deliberative and predecisional, so it must not be disclosed or released by reviewers. Because the manuscript has not yet been approved for publication by the U.S. Geological Survey (USGS), it does not represent any official USGS finding or policy.” was erroneously published in the Acknowledgments section. The paragraph should have been removed when the manuscript was accepted for publication. The publisher apologies for the error.
